# Determination of the Residual Anthracene Concentration in Cultures of Haloalkalitolerant Actinomycetes by Excitation Fluorescence, Emission Fluorescence, and Synchronous Fluorescence: Comparative Study

**DOI:** 10.1155/2016/6287931

**Published:** 2016-01-26

**Authors:** Reyna del Carmen Lara-Severino, Miguel Ángel Camacho-López, Jessica Marlene García-Macedo, Leobardo M. Gómez-Oliván, Ángel H. Sandoval-Trujillo, Keila Isaac-Olive, Ninfa Ramírez-Durán

**Affiliations:** ^1^Facultad de Medicina, Universidad Autónoma del Estado de México, 50180 Toluca, MEX, Mexico; ^2^Facultad de Química, Universidad Autónoma del Estado de México, 50120 Toluca, MEX, Mexico; ^3^Departamento de Sistemas Biológicos, Universidad Autónoma Metropolitana-Xochimilco, 04960 México, DF, Mexico

## Abstract

Polycyclic aromatic hydrocarbons (PAHs) are compounds that can be quantified by fluorescence due to their high quantum yield. Haloalkalitolerant bacteria tolerate wide concentration ranges of NaCl and pH. They are potentially useful in the PAHs bioremediation of saline environments. However, it is known that salinity of the sample affects fluorescence signal regardless of the method. The objective of this work was to carry out a comparative study based on the sensitivity, linearity, and detection limits of the excitation, emission, and synchronous fluorescence methods, during the quantification of the residual anthracene concentration from the following haloalkalitolerant actinomycetes cultures* Kocuria rosea, Kocuria palustris, Microbacterium testaceum*, and 4 strains of* Nocardia farcinica*, in order to establish the proper fluorescence method to study the PAHs biodegrading capacity of haloalkalitolerant actinobacteria. The study demonstrated statistical differences among the strains and among the fluorescence methods regarding the anthracene residual concentration. The results showed that excitation and emission fluorescence methods performed very similarly but sensitivity in excitation fluorescence is slightly higher. Synchronous fluorescence using Δ*λ* = 150 nm is not the most convenient method. Therefore we propose the excitation fluorescence as the fluorescence method to be used in the study of the PAHs biodegrading capacity of haloalkalitolerant actinomycetes.

## 1. Introduction

Polycyclic aromatic hydrocarbons (PAHs) are a group of highly toxic and persistent organic pollutants that are widely distributed in the environment worldwide [1]. PAHs are ubiquitous, very stable in the environment [2]. They are potentially dangerous due to their carcinogenicity and mutagenicity [3]; thus, their removal is an issue of great interest.

Microbial biodegradation is an attractive option for PAHs removal. The PAHs degradation can be quantified by luminescence techniques such as fluorescence, due to their high quantum yield [4] and relatively easy analysis, since fluorescence methods do not require extensive pretreatment of the sample and can be used with conventional instrumentation (spectrofluorometer).

One of the compounds used as model in studies of PAHs degradation by microorganisms is anthracene, a polycyclic aromatic hydrocarbon of three rings, which has been listed as one of the priority environmental pollutants by the United States Environmental Protection Agency. There is insufficient information to classify the anthracene as a substance that causes cancer (http://www.epa.gov/osw/hazard/wastemin/priority.htm) but causes concern due to the fact that their structure is resemblant to carcinogenic PAHs such as benzo[a]pyrene and benzo[a]anthracene [5]. Therefore, anthracene is usually used as a PAH model in studies of degradation [6–8].

Traditional fluorescence collects a spectrum by scanning the excitation wavelength (*λ*_exc_) while the emission wavelength is fixed (*λ*_em_) (excitation spectrum). The emission spectrum is obtained in a similar manner; in this case, the excitation wavelength is constant (*λ*_exc_), and the emission wavelength is scanned (*λ*_em_). This method is known as excitation-emission fluorescence or excitation fluorescence and emission fluorescence. Both techniques are extensively used for individual PAHs analysis due to their high sensitivity, selectivity, speed, and relatively low cost [9, 10]. This fluorescence method is not useful in the multicomponent analysis of PAHs because these compounds have similar structures and there is overlapping of the spectral bands, which are generally wide [11].

Synchronous fluorescence is another method of fluorescence spectroscopy that emerged in 1971 [11]. In one of its variants, the excitation and emission monochromators are scanned simultaneously with a constant Δ*λ* between them. It has been reported that this technique increases the selectivity (the broad excitation-emission fluorescence bands are better defined) and maintains the high sensitivity [12], resulting in the possibility of analysing multicomponent samples (which surpasses conventional excitation-emission fluorescence method). This is the main advantage and the reason for its increasing popularity.

The bioremediation of PAHs contaminated environments is possible with halotolerant or alkalitolerant actinomycetes such as* Arthrobacter crystallopoietes*,* Arthrobacter arilaitensis*,* Micrococcus* sp.,* Dietzia* sp., and* Rhodococcus* sp. [13, 14]. However, there are no reports about the use of haloalkalitolerant actinomycetes (polyextremophile microorganisms able to live in either the absence or presence of salt, and in presence of pH values in the range 8–11) which were able to grow in PAHs polluted environments. The use of these microorganisms in bioremediation processes is more convenient because unlike halophilic or alkalophilic ones (which grow at a specific NaCl and pH value), haloalkalitolerant grow within a range of NaCl and pH.

A general method for studying the PAHs bioremediation capacity of haloalkalitolerant actinomycetes is by preparing culture media containing PAHs and measuring the residual anthracene concentration in the cultures, by fluorescence, for instance, at different times. One of the culture media used to grow bacteria, when fluorescence measurements are going to be carried out, is the minimal salt medium (MSM) because it is considered a transparent medium from the fluorescence point of view [15–17]. However, these haloalkalitolerant actinomycetes require NaCl concentration for growing, and it is already known that fluorescence signal is affected by the salinity of the sample regardless of the fluorescence method employed.

The main question of this work is as follows: Would it be adequate to use excitation fluorescence, emission fluorescence, or synchronous fluorescence to study the biodegrading capacity of haloalkalitolerant actinomycetes requiring high NaCl concentration for their growth (halotolerant bacteria)? And the objective is carrying out a comparative study based on the sensitivity, linearity, and detection limits of the excitation, emission, and synchronous fluorescence methods during the quantification of the residual anthracene concentration from haloalkalitolerant cultures in order to establish the most proper fluorescence method to study the PAHs biodegrading capacity of these kind of bacteria.

## 2. Material and Methods

### 2.1. Strains

Seven strains of haloalkalitolerant actinomycetes from soda soils were studied. These strains had been already identified by the 16S rRNA sequence. They tolerate %NaCl and pH ranges as follow:* Kocuria palustris* (0–25%; pH 5–12),* Kocuria rosea* (0–10%; pH 5–11);* Microbacterium testaceum* (0–10%; pH 5–11); and four strains of* Nocardia farcinica* (0–3%; pH 5–10). However their optimal growth conditions are as follows:* Kocuria palustris* (10% NaCl);* Kocuria rosea* and* Microbacterium testaceum* (3% NaCl); and four strains of* Nocardia farcinica* (0.5% NaCl). For all strains the optimal pH was 8. In this work the haloalkalitolerant actinomycetes were always cultivated at their optimal conditions for guaranteeing their best performance.

Nucleotide sequence data of these strains are available in GenBank database under the accession numbers from KP100512 to KP100518.

### 2.2. Culture Media

Minimal salt medium (MSM) was prepared according to Sang et al. [17]: (NH_4_)_2_SO_4_, 1000 mg/L; Na_2_HPO_4_, 800 mg/L; K_2_HPO_4_, 200 mg/L; MgSO_4_·7H_2_O, 200 mg/L; CaCl_2_·2H_2_O, 100 mg/L; FeCl_3_·H_2_O, 5 mg/L; (NH_4_)_6_Mo_7_O_24_·H_2_O, 1 mg/L. Three variants of this medium were prepared with different NaCl concentrations (0.5%, 3%, and 10%) for optimal grow of each strain. These media were called MSM-0.5%, MSM-3%, and MSM-10%. Each solution was adjusted to pH 8 and sterilised in an autoclave at 121°C for 15 minutes.

These MSM media were divided in two sets; one set was the three MSM media already prepared and to the second one anthracene was added as follows. An initial stock solution of 54 *μ*g/mL of anthracene [2.7 mg of solid anthracene (Fluka) dissolved in acetone] was prepared in an amber-colour volumetric flask. An aliquot of 0.5 mL of this solution was transferred to three 100 mL of these flasks, and MSM-0.5%, MSM-3%, and MSM-10% were, respectively, filled to the mark. These three 0.27 *μ*g/mL anthracene media were called ANTH-0.5%, ANTH-3%, and ANTH-10%, respectively. They were sterilised by membrane filtration (Millipore filter, Millex with a 0.22 *μ*m Durapore membrane, SLGV033RS).

### 2.3. Inoculation of MSM and ANTH Media

From previous studies it is known that the exponential growth phase of these strains in MSM media concludes at 7.5 h for all strains, except* Kocuria palustris*, which concludes at 10 h. Therefore, the inocula in this work were collected at 4 and 5 h, respectively.

Volumes of each inoculum equivalent to a OD_600 nm_ = 0.25 [0.5 Nephelometric Turbidity Units (NTU)] were transferred to 60 mL of the corresponding ANTH and MSM media, contained in screw cap Erlenmeyer flasks. In order to avoid the light, the Erlenmeyer flasks were covered with aluminium foil. The inoculated media were incubated at room temperature and 150 rpm during 48 h.

### 2.4. Sample Preparation for Fluorescence Analyses

#### 2.4.1. Samples Matrices

After 1, 24, and 48 h of incubation, 5 mL of each MSM cultures was centrifuged (10,000 rpm, 5 minutes, room temperature) for supernatant and biomass separation. These supernatants were analysed by excitation, emission, and synchronous fluorescence (as described in Section 2.7) to investigate whether the strains produced any metabolite that could interfere with the anthracene fluorescence signal. Other samples also analysed by this three fluorescence method were empty cuvette, distilled water, MSM-0.5%, MSM-3%, and MSM-10%. All these spectra make up the matrices signals; therefore there is a matrix signal for each NaCl concentration in each fluorescence method. Each experiment was performed five times.

#### 2.4.2. Control Samples

Five millilitres of the ANTH-0.5%, ANTH-3%, and ANTH-10% media without inoculation was analysed by the three fluorescence methods (Section 2.7) at 0, 1, 24, and 48 h to monitor any possible anthracene loss by evaporation, sublimation, or photodegradation process. Each experiment was performed five times.

#### 2.4.3. Samples for Anthracene Quantification from Strains Culture

After 1, 24, and 48 h of incubation, 5 mL of each ANTH cultures was centrifuged (10,000 rpm, 5 minutes, room temperature) for supernatant and biomass separation. The residual anthracene concentration was determined in each supernatant by excitation, emission, and synchronous fluorescence (as described in Section 2.7). The initial concentration of anthracene (*t* = 0) corresponds to the anthracene concentration of ANTH media without inoculation (control samples). Each experiment was performed five times (five independent inoculations).

### 2.5. Determination of Sensitivity and Linearity

One mL of the 54 *μ*g/mL anthracene stock solution was transferred to three 100 mL amber-colour volumetric flasks covered, and MSM-0.5%, MSM-3%, and MSM-10% were, respectively, filled to the mark. These 540 ng/mL anthracene standard solutions were called CC-0.5%, CC-3%, and CC-10%, respectively. They were later used in the constructions of the anthracene calibration curves.

From the CC-0.5%, CC-3%, and CC-10% standard solutions, the volumes necessary to prepare 3 mL of 360, 270, 180, 90, 45, and 22.5 ng/mL anthracene standard solutions were taken, respectively. Each solution was completed to 3 mL volume using the corresponding MSM-0.5%, MSM-3%, and MSM-10% media, transferred to the spectrofluorometer cuvette followed by the acquisition of the excitation, emission, and synchronous fluorescence spectra. The conditions for register of the spectra are detailed in Section 2.7.

Each fluorescence intensity was corrected by the inner-filter effect using the Absorbance-Based Approach (ABA) [18, 19]. This approach uses the measured absorbance (*Aλ*) at each pair of excitation (*λ*_exc_) and emission (*λ*_em_) wavelengths to convert the observed fluorescence intensity (*F*_obs_) into the corrected fluorescence intensity (*F*_corr_) according to the following equation:

(1) $$\begin{array}{c} F_{\text{corr}}=F_{\text{obs}}\times \text{antilog}\left[\;\frac{\left(A_{\lambda_{\text{exc}}}+A_{\lambda_{\text{em}}}\right)}{2}\;\right]. \end{array}$$

The absorbance measurements were carried out in a spectrophotometer (Perkin-Elmer UV-Vis, model 551S), using 1 cm quartz cuvette. The absorbance spectrum was recorded from 300 to 500 nm.

From these corrected fluorescence intensities (*F*_corr_), three calibration curves (fluorescence intensity versus anthracene concentration) were built for each fluorescence method. These nine curves were fitted to a straight line using the weighted least-squares model, and the slopes of these lines are the sensitivities of the methods for determining anthracene under the three different %NaCl employed. Each curve was built as the average curve of three repetitions. The significant test for the intercept was carried out. The linearity was evaluated by the determination coefficient (*R* ^2^) and the regression coefficient (*r* ^2^).

### 2.6. Determination of the Detection Limit

The detection limits for each %NaCl condition in each fluorescence method was calculated according to the equation: DL (ng/mL) = [(Maximal Background Signal) + 3 *∗* (Standard deviation of the background signal)/Sensitivity].

The maximal background signal for each fluorescence method and NaCl concentration was taken from the set of spectra that makes up each matrix signal (Section 2.4.1). In Section 2.4.1 it was described that each measurement was repeated 5 times; therefore the standard deviation of the background signal was calculated from there. The sensitivity was determined as described above (Section 2.5). Each experiment was performed five times.

### 2.7. Acquisition of Fluorescence Spectra

The fluorescence spectra of the empty cuvette, distilled water, MSM media, standard solution of each CC calibration curve, inoculated MSM media (matrix) at 1, 24, and 48 h, inoculated ANTH media (samples) at 1, 24, and 48 h, and the ANTH media at 0, 1, 24, and 48 h (control samples) were scanned by a spectrofluorometer (Horiba, Fluoromax-3) at room temperature. The fluorescence measurements were performed using a 1 × 1 cm standard quartz cell. The conditions for spectra collection in the three methods (excitation, emission, and synchronous) were the following:

(i) Excitation fluorescence spectra (230–390) nm: emission wavelength of 400 nm, resolution of 2 nm, integration time = 0.5 s, and slits of 0.5 mm.
(ii) Emission fluorescence spectra (365–500) nm: excitation wavelength of 340 nm, resolution of 2 nm, integration time = 0.5 s, and slits of 2 nm.
(iii) Synchronous fluorescence spectra (200–450) nm: Δ*λ* = 150 nm, resolution of 2 nm, integration time = 0.5 s, and slits of 2 nm.

From the fluorescence signal of the matrix and CC standard solutions, the emission wavelength, excitation wavelength, and synchronous fluorescence wavelength of anthracene were selected for each %NaCl employed.

### 2.8. Statistical Comparison

The relative concentrations of residual anthracene from the ANTH media determined by the three methods (excitation, emission, and synchronous) after 1 h of strain inoculation were statistically compared by a bivariate analysis. The two studied variables were strains (7 levels) and fluorescence method (3 levels).

## 3. Results and Discussion

### 3.1. Selection of the Excitation Wavelength for the Acquisition of Emission Spectra

**Figure 1:**
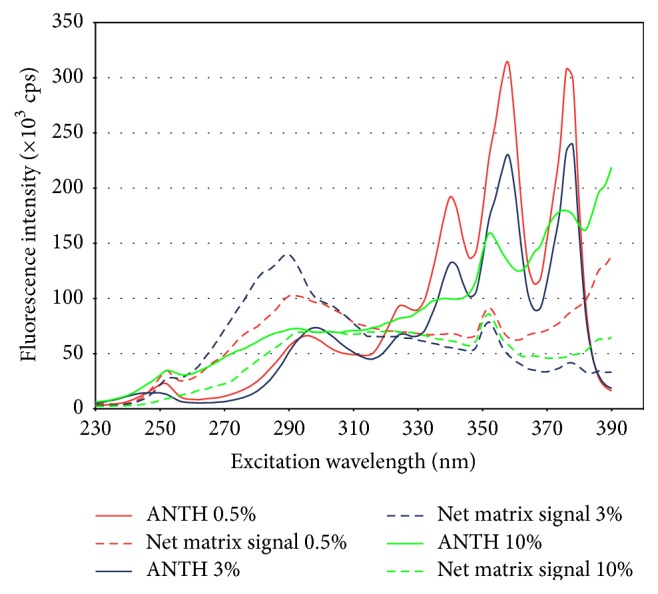
Excitation spectra with *λ*_emission_ = 401 nm of the 22.5 ng/mL anthracene solutions and the net matrix signal fluorescence intensity.

Figure 1 shows the excitation spectra of the net background signal, and of the three diluted anthracene standard solutions (22.5 ng/mL). The emission wavelength was set at 400 nm because the literature reports that anthracene has an emission band around this value. The net background signal in this figure is composed by those fluorescence signals from the matrix which have a maximal signal in some region of the whole scan. The ideal excitation wavelength is the one that selectively excites the analyte, maximally reducing the excitation of the matrix components (background signal). The figure shows that 340 nm, 352 nm, 358 nm, and 374–378 nm bands could be employed as excitation wavelength.

Two of the excitation *λ*s of anthracene (352 and 358 nm) are very close or coincide with the 352 nm excitation *λ* of the matrix components (background signals); consequently, this *λ* is not adequate for excitation of the sample because it also excites the matrix. A similar finding is obtained with the excitation *λ* that appears at 374 nm, 376 nm, and 378 nm; some matrix components are excited at these wavelengths. The ideal excitation situation only occurs with the excitation *λ* of 340 nm for the three anthracene solutions prepared in different %NaCl. Therefore, this excitation *λ* was used to obtain the emission fluorescence spectra of all analysed samples in this work.

### 3.2. Selection of the Emission Wavelength for the Acquisition of Excitation Spectra

**Figure 2:**
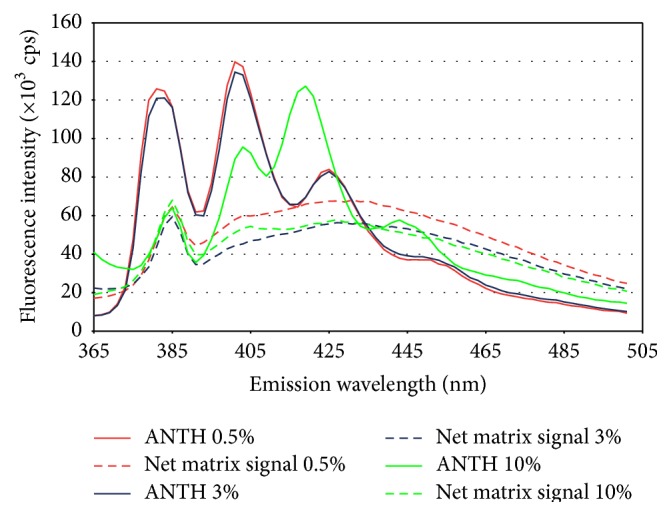
Emission spectra with *λ*_excitation_ = 340 nm of the 22.5 ng/mL anthracene solutions and the net matrix signal fluorescence intensity.

Figure 2 shows the emission spectra of the net background signal, and of the three diluted anthracene standard solutions (22.5 ng/mL). The excitation wavelength was set at 340 nm as explained above. The net background signal in this figure is composed of those fluorescence signals from the matrix which have a maximal signal in some region of the whole scan. The ideal emission wavelength is defined as the wavelength where the analyte emission spectrum has the band with lowest contribution of the matrix components (background signal). The figure shows that 380 nm, 401–403 nm, and 419–425 nm bands could be employed as emission wavelength. From them the 401–403 nm is the wavelength range where the anthracene has an intense emission band while the matrix has a minimal emission. Therefore, the band at 401 nm was selected for obtaining the subsequent excitation spectra.

### 3.3. Selection of Δ*λ* for Acquiring the Synchronous Fluorescence Spectra

**Figure 3:**
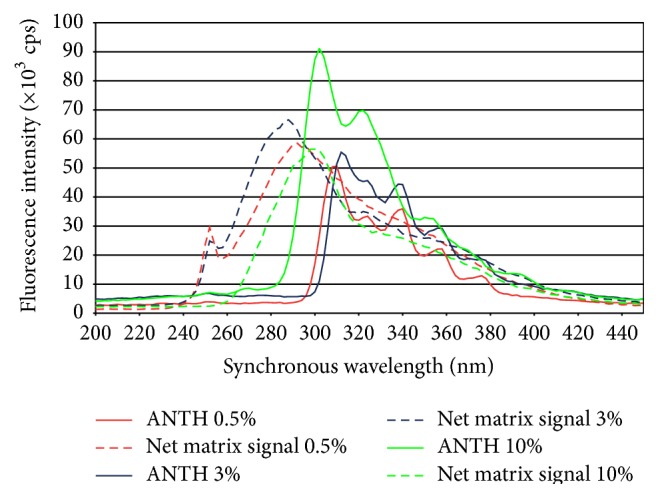
Synchronous spectra with Δ*λ* = 150 nm of the 180 ng/mL anthracene solutions and the net matrix signal fluorescence intensity.

In this work, Δ*λ* = 150 nm was used to acquire the synchronous fluorescence spectra of anthracene because Cai et al. [12] experimentally demonstrated that this value of Δ*λ* is optimal for obtaining good sensitivity and selectivity.  Figure 3 shows the synchronous fluorescence spectra of the net background and the 180 ng/mL anthracene standard solutions under this condition. As in the previous cases, the net background signal in this figure is composed of those fluorescence signals from the matrix which have a maximal signal in some region of the whole scan spectra.

### 3.4. Selection of the Excitation Band for Quantifying Anthracene by Excitation Fluorescence

Figure 1 also displays the signals of the 22.5 ng/mL anthracene standard solutions prepared from ANTH-0.5%, ANTH-3%, and ANTH-10%. The only difference among these solutions is the NaCl concentration (%) present. If there is no effect of the NaCl concentration on the excitation fluorescence signal, it is expected that there are no differences in the (i) shape, (ii) position, and (iii) intensity of the bands from these solutions.

(i) Shape: this parameter in the three excitation spectra was maintained. (ii) Position: the lower wavelength bands (252 nm, 294 nm, 324 nm, and 340 nm) coincide in position for the three solutions. The bands at higher wavelength are slightly shifted (taking as reference the 0.5% spectrum); 358 nm band is present in the 0.5% and 3% NaCl media but is shifted to 352 nm in the 10% NaCl medium. The 376 nm band appears in the 0.5% medium; in the 3% medium, it is located at 378 nm, and in the 10% medium, it appears at 374 nm. (iii) Intensity: anthracene bands in 0.5% are more intense than those in 3%, and both are much more intense than those in 10% (340 nm band in 10% NaCl is 50% less intense than that in 0.5% NaCl).

To quantify anthracene in saline solutions the 340 nm band is very convenient because its position is not shifted with salinity. However, high %NaCl reduces band intensity; therefore the final selection depends on the concentration to measure and the background signal. In this work, to quantify anthracene by excitation fluorescence, the 340 nm band was used for samples in 0.5% and 3% of NaCl and the 378 nm band was employed for samples in 10% NaCl.

### 3.5. Selection of the Emission Band for Quantifying Anthracene by Emission Fluorescence

Figure 2 presents the emission spectra corresponding to the same samples presented in Figure 1, all excited at 340 nm. As in the previous case, the (i) shape, (ii) position, and (iii) intensity of the band spectra are analysed. (i) There are no changes in the shape of the emission spectra; this means that the number of emission bands is the same in all spectra. (ii) There is not shifting in the anthracene emission bands in the 0.5% and 3% NaCl media. The three emission maxima are at 381 nm, 401 nm, and 425 nm. The position of these maxima is shifted in the 10% NaCl medium, the maxima are at 385 nm, 403 nm, and 419 nm. (iii) Band intensities are similar for anthracene solutions prepared in 0.5% and 3% NaCl media (425 nm ≪ 381 nm < 401 nm) but different for the 10% media (385 nm < 403 nm ≪ 419 nm).

The ideal situation for quantifying anthracene by emission fluorescence is to choose an emission *λ* at which the matrix components have little or no emission and the anthracene has an intense band. Therefore, the quantification of anthracene in the ANTH-0.5% and ANTH-3% media was performed with the 401 nm band, which was only slightly affected by the matrix signal and had a good intensity. In the case of samples in ANTH-10% the band used was 419 nm.

### 3.6. Selection of Emission Bands for Anthracene Quantification by Synchronous Fluorescence

Figure 3 displays the synchronous fluorescence spectra with Δ*λ* = 150 nm of the 180 ng/mL anthracene standard solution prepared from CC-0.5%, CC-3%, and CC-10%, as well as the net background spectra. It can be observed that the shape of the spectrum of the anthracene from CC-0.5% and CC-3% standard solutions is very similar but varies in the CC-10% solution. The first two spectra have more bands than the last one. Band intensity in this fluorescence method behaves in contrast to fluorescence excitation and emission. In this case, band intensity increases with the increasing NaCl concentration.

**Figure 4:**
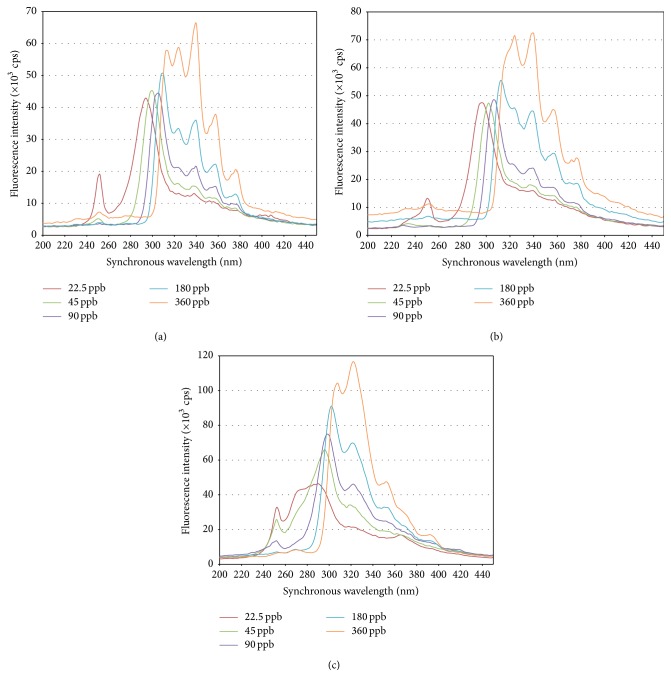
(a) Synchronous fluorescence spectra of various anthracene standard solutions at different concentrations prepared in the MSM-0.5% medium. (b) Synchronous fluorescence spectra of various anthracene standard solutions at different concentrations prepared in the MSM-3% medium. (c) Synchronous fluorescence spectra of various anthracene standard solutions at different concentrations prepared in the MSM-10% medium.

In contrast with the excitation and emission fluorescence spectra of anthracene, which are well known, the synchronous fluorescence spectrum varies with the instrumental conditions used during acquisition (Δ*λ*), and some bands are influenced in a greater or lesser degree by the solvent. In order to determine the bands that proportionally change and do not vary their position with the anthracene concentration, the synchronous fluorescence spectra of the standard solutions prepared from CC-0.5%, CC-3%, and CC-10% were graphed in Figure 4.

From Figure 4 it can be noticed that the 22.5 ng/mL anthracene standard solutions in the three media under study have the 250 nm band, but it gradually decreases until disappearance with an increasing anthracene concentration, suggesting that it is associated with or very influenced by the medium. Given that the only difference between the three ANTH media is the NaCl concentration, then the intensity of this band is influenced by the medium salinity. Our initial idea was to use this band for quantification, because Cai et al. [12] demonstrated that at Δ*λ* = 150 nm it is optimal for obtaining good sensitivity and selectivity. In our case however, the 250 nm band was not useful probably because although our matrix is aqueous like the one they used, it is not exactly the same.

A decision had to be made regarding which signal of the synchronous fluorescence spectrum to use for the anthracene quantification.  Figure 4(a) shows bands at 296 nm, 300 nm, 306 nm, 310 nm, and 314 nm which correspond to anthracene concentrations (ng/mL) 22.5, 45, 90, 180, and 360, respectively. They could be considered shifting of the same band, but there is not proportionality between band intensity and anthracene concentration; therefore they should be associated with the solvent. This same effect is observed in Figure 4(b) with the 300 nm band corresponding to the 22.5 ng/mL concentration, which shifts with the increased anthracene concentration to reach a value of 316 nm for 360 ng/mL. This feature is also observed in Figure 4(c) with the 294 nm band, corresponding to the 22.5 ng/mL concentration, which shifts as the anthracene concentration increases up to 308 nm at a concentration of 360 ng/mL. None of these bands are useful for quantifying anthracene.

Figure 4 shows a band at 322–324 nm in the three media under study; this band does not shift with an increasing anthracene concentration, and its intensity varies proportionally with the concentration. Therefore, this band is useful for anthracene quantification. In the spectra corresponding to the ANTH-0.5% and ANTH-3% media, the same behaviour described previously is observed with the bands at 340 nm, 358 nm, and 378 nm. The spectra of anthracene in the ANTH-10% medium do not present the bands at 340 nm and 378 nm, and the 358 nm band is shifted to 352 nm. This difference between the spectra is attributed to the different concentrations of NaCl present in the three media. As the 340 nm band is most intense for the ANTH-0.5% and ANTH-3% media and the 322 nm band is most intense for the ANTH-10% medium these bands were chosen for anthracene quantification by synchronous fluorescence.

### 3.7. Sensitivity, Linearity, and Detection Limit

The absorbance measured for all anthracene standard solutions at their respective excitation and emission wavelengths were in the range 0.041–0.066, and the ratio *F*_obs_/*F*_corr_ was 0.9 for the three fluorescence methods. We do not attribute this attenuation in the fluorescence signal to an inner-filter effect because the correction factor was the same in all solutions, and this value changes in the inner-filter effect, being higher at higher concentrations [19]. Moreover, our calibration curves were linear in the whole concentration range. The maxima absorbance value was slightly over 0.06, being the inner-filter effect negligible. The corrected fluorescence values from the anthracene standard solutions give a calibration curve as linear as the one obtained from the observed values, demonstrating an attenuation effect but not inner-filter effect. Since this attenuation effect is present in all samples equally, it does not affect quantification.

Table 1 shows the calibration curves obtained from a weighted regression using the least square model. The sensitivities are the slope of the curves, and ideally they should have the same value for the ANTH-0.5%, ANTH-3%, and ANTH-10% media, within the same fluorescence method. Sensitivities values were always different for the three media in the three analysed methods. This result corroborates the salinity effect on the fluorescence mechanism. Among the methods, excitation fluorescence is 1.1–1.3 times more sensitive than emission, and 22–52 times more than synchronous fluorescence. These results agree with other studies reporting that excitation fluorescence and emission fluorescence are extremely sensitive and that synchronous fluorescence is less sensitive but more selective [11]. In our case, however, synchronous fluorescence was not more selective, because band definition was better in excitation and emission fluorescence.

From the sensitivities values, it seems that excitation fluorescence is the one less affected by salinity. The sensitivity reduction from ANTH-0.5% to ANTH-10% was 20%. In emission fluorescence, this reduction was 30%; and unexpectedly in synchronous the sensitivity value was not reduced but increased in 188%.

Calibration curves for excitation and emission fluorescence do not have statistically significant intercept at 95%, meaning that there is not a matrix effect on the analyte fluorescence signal. This is a very important point for a quantification method. In synchronous fluorescence however, the intercept was always statistically significant, meaning that at zero analyte concentration there is a fluorescence signal attributed to the matrix. Therefore in this study under our conditions the matrix, unlike excitation and emission fluorescence, has effect on the synchronous signal. The intercept was not constant for the three ANTH media, indicating that NaCl concentration is also contributing to matrix effect.

Table 1 also shows the determination coefficient and the linear regression coefficient. The first one indicates how well the model fits the data. In our case, the model is a straight line. It is a measure that allows us to determine how certain one can be in making predictions from a certain model/graph. The second one measures the strength and direction of the linear relationship between two variables. In this case, both coefficients are higher than 0.997, indicating that a straight line is a very adequate model to fit our data (anthracene concentration* versus* fluorescence intensity) for the three fluorescence methods in the concentration range studied and that this linear relationship is strong and positive (increasing the concentration increases the fluorescence signal). In addition, the dilution factor among the standard solutions employed in the calibration curves (1 : 16, 1 : 8, 1 : 4, 1 : 2, 1 : 1.3) coincides with the reduction in the fluorescence intensity. Therefore, linearity is assured in the anthracene determination in all cases for all methods in the concentration range studied.

Table 1 also shows the detection limits for anthracene in the three media studied by the three fluorescence methods. The detection limit is an order of magnitude lower in excitation and emission fluorescence than in synchronous fluorescence because the net maximal matrix signal does not vary in intensity in the three methods at the wavelengths selected for quantification (Figures 1–3), while the anthracene sensitivity decreases between 20 and 50 times from excitation-emission fluorescence to synchronous fluorescence.

### 3.8. Quantification of the Residual Anthracene Concentration in Actinomycete Cultures by Emission Fluorescence, Excitation Fluorescence, and Synchronous Fluorescence

Thus, for ensuring the best accuracy, all solutions were prepared in the same manner, using the same reagents and matrix solution and the same preparation techniques, and measured at the same temperature after the same amount of time.

The fluorescence intensity of the ANTH solutions (control solutions) measured at 0, 1, 24, and 48 h by the three methods showed a relative standard deviation (RSD) less than 5%, indicating that the incubation time does not affect the fluorescence signal of anthracene and no evaporation, sublimation, or photolysis processes were involved during sampling handling.

The capabilities for biodegrading PAHs of the genera to which the strains analysed belong have been already reported [20–23]. The residual anthracene values determined in this study are in the range of those reported for other PAHs (naphthalene, phenanthrene, and dihydrophenanthrene) for the study genera [20–22]. Therefore, the decrease in the anthracene fluorescence signal of the samples studied in this work is attributed to the consumption of anthracene by the strains.

Table 2 lists the values of the initial anthracene concentration and the residual anthracene present in the actinomycetes culture at 0, 1, 24, and 48 h by the three methods. According to the results shown in the table, all strains were capable of significantly transforming anthracene during the first hour. During the intervals 1–24 h and 24–48 h, this transformation was lower.

From Table 2 the precision in the anthracene concentration can be calculated (%RSD = SD/Mean *∗* 100). This parameter is better in excitation > emission > synchronous fluorescence. A bivariate analysis showed statistical differences (*p* < 0.05) for the fluorescence methods and for the strains. Statistical differences among strains mean that the biodegrading capacity of all these strains is not alike. Statistical differences among the methods indicate that the performance of the three fluorescence methods is not statistically similar. These differences are specifically between excitation and synchronous and emission and synchronous. There is not statistical difference between excitation and emission. Other reports have demonstrated that results obtained by excitation and emission fluorescence (in the PAHs determination) do not differ from results obtained by other techniques such as gas chromatography or HPLC [24].

## 4. Conclusions

Fluorescence is a very convenient method to study PAHs biodegradation because it is simple, easy, fast, inexpensive, very sensitive, and reproducible. For these reasons fluorescence was our method of choice for studying the capabilities of haloalkalitolerant actinomycetes for degrading PAHs. These bacteria require NaCl for growing; therefore our study was focussed to investigate the performance of excitation, emission, and synchronous fluorescence in the determination of anthracene in the presence of different NaCl concentrations in order to stablish the most proper fluorescence method to study the PAHs biodegrading properties of these bacteria.

Our results show that anthracene excitation fluorescence spectrum in MSM (called ANTH) does not change in shape, relative band intensity, or band position with the increasing %NaCl. The calibration curves for the three NaCl concentrations indicate that there is not background signal in the fluorescence response (equation does not have a statistically significant *y* intercept); the sensitivity is very high, and it is only reduced 20% from the medium containing 10% NaCl respective to the one with 0.5% NaCl; the straight line is a very good fitting model for the calibration curves (*R* ^2^ > 0.998); and fluorescence signal* versus* concentration is linear in the studied range (22.5–360 ng/mL). The inner-filter effect present in the fluorescence signal of the three calibration curves is negligible. The detection limit obtained, by using culture without anthracene as matrix background, is 11, 13, and 20 ng/mL for the media with 0.5%, 3%, and 10% NaCl, respectively.

In the case of the anthracene emission fluorescence spectra in MSM and different NaCl concentration, most of the features found in excitation fluorescence are similar with the following differences: the spectrum does not change in shape or band position but the relative band intensity changes, and the sensitivity is reduced 30% when NaCl concentration is increased from 0.5% to 10%.

The synchronous fluorescence spectra features of anthracene contained in MSM with different NaCl concentrations quite differ from the previous cases. The spectrum changes the shape and band position with the increasing NaCl concentration. The *y* intercept in the calibration curves was statistically significant; therefore at zero anthracene concentration there is a fluorescence signal most likely coming from the matrix. Unlike excitation and emission spectra where the sensitivity was reduced with the increasing NaCl concentration, in synchronous fluorescence the sensitivity was increased 188% when NaCl concentration was increased from 0.5% to 10%. This result clearly shows that, in this fluorescence method, under the conditions used in this work the anthracene signal is overestimated due to matrix contribution. Detection limits are worse than those from excitation and emission methods, being one order higher. Selectivity was not improved in the anthracene determination using Δ*λ* = 150 nm, in comparison to excitation and emission fluorescence.

Despite the fact that the three fluorescence methods indicate anthracene biodegradation, the results described above demonstrated that synchronous fluorescence is not the most adequate method (among fluorescence methods) to determining anthracene in haloalkalitolerant actinomycetes cultures with NaCl concentrations as high as 10%, when Δ*λ* = 150 nm is used. Although excitation and emission fluorescence behave very similarly, excitation fluorescence performs slightly better, being our proposed fluorescence method for determining the residual anthracene concentration present in cultures of haloalkalitolerant actinomycetes potentially degraders of PAHs, as long as standards and samples are prepared exactly in the same manner.

## Figures and Tables

**Table 1:** Calibration curves and detection limits for the anthracene determination by excitation, emission, and fluorescence methods.

	Ant medium	*λ* (nm)	Calibration curve	Determination coefficient (*R* ^2^)	Regression coefficient (*r*)	Detection limit (ng/mL)
Excitation fluorescence	0.5%	340	*y* = 7821*x*	0.998	0.999	13
	3%	340	*y* = 6704*x*	0.999	0.999	11
	10%	370	*y* = 6308*x*	0.997	0.998	20

Emission fluorescence	0.5%	401	*y* = 6928*x*	0.999	0.999	13
	3%	401	*y* = 6074*x*	0.999	0.999	13
	10%	419	*y* = 4879*x*	0.999	0.999	10

Synchronous fluorescence	0.5%	340	*y* = 150*x* + 8438	0.998	0.999	155
	3%	340	*y* = 136*x* + 12683	0.998	0.999	112
	10%	322	*y* = 283*x* + 17089	0.999	0.999	184

**Table 2:** Anthracene residual concentration in the cultured media inoculated with haloalkalitolerant actinomycetes by the three fluorescence methods.

Strains	Anthracene residual concentration (ng/mL) (*n* = 5)			
	Time (h)	Excitation fluorescence	Emission fluorescence	Synchronous Fluorescence
All actinomycetes	0	270 ± 10	270 ± 8	270 ± 8

*Nocardia farcinica 1*	1	145 ± 4	139 ± 7	206 ± 27
	24	132 ± 2	130 ± 9	156 ± 7
	48	132 ± 3	128 ± 13	155 ± 11

*Nocardia farcinica 2*	1	152 ± 8	153 ± 8	170 ± 21
	24	137 ± 7	133 ± 11	125 ± 15
	48	134 ± 7	132 ± 20	125 ± 14

*Nocardia farcinica 3*	1	142 ± 8	132 ± 9	184 ± 6
	24	139 ± 3	136 ± 11	142 ± 5
	48	141 ± 7	129 ± 10	143 ± 8

*Nocardia farcinica 4*	1	149 ± 8	143 ± 15	170 ± 18
	24	149 ± 7	134 ± 6	148 ± 11
	48	138 ± 3	131 ± 6	143 ± 6

*Kocuria rosea*	1	155 ± 3	153 ± 5	242 ± 12
	24	149 ± 5	150 ± 8	215 ± 16
	48	143 ± 5	137 ± 10	214 ± 7

*Microbacterium testaceum*	1	173 ± 7	163 ± 13	213 ± 10
	24	161 ± 4	156 ± 8	213 ± 8
	48	156 ± 2	145 ± 15	212 ± 18

*Kocuria palustris*	1	137 ± 7	121 ± 18	189 ± 7
	24	128 ± 6	123 ± 11	180 ± 9
	48	119 ± 5	123 ± 10	180 ± 3

Value reported as mean ± SD (*n* = 5).
